# Identification and Phylogenetic Characterization of Cobalamin Biosynthetic Genes of *Ensifer adhaerens*

**DOI:** 10.1264/jsme2.ME12069

**Published:** 2012-12-19

**Authors:** Hoan Thi Vu, Hideomi Itoh, Satoshi Ishii, Keishi Senoo, Shigeto Otsuka

**Affiliations:** 1 Department of Applied Biological Chemistry, Graduate School of Agricultural and Life Sciences, The University of Tokyo, 1–1–1 Yayoi, Bunkyo-ku, Tokyo 113–8657, Japan

**Keywords:** cobalamin biosynthetic genes, *cob*, *Ensifer*, *Sinorhizobium*, phylogeny

## Abstract

*Ensifer adhaerens* CSBa was screened as a cobalamin producer. The draft genome sequence revealed that the strain possesses 22 cobalamin biosynthetic genes (*cob* genes). The *cob* gene arrangement on the genome of *E. adhaerens* CSBa was similar to that of other *Ensifer* species, and most similar to that of *Pseudomonas denitrificans* SC510. The *cobN* sequence phylogeny was generally congruent with that of the 16S rRNA gene, and it is suggeted that *E. adhaerens* CSBa might have inherited the *cob* genes from common ancestors of the *Ensifer* species. It was also suggested that the *cob* genes can be laterally transferred.

Cobalamin (vitamin B_12_) is one of the most structurally complex, nonpolymeric biomolecules biosynthesized only by bacteria and archaea (2, 12, 15). Today, cobalamin is industrially produced exclusively by biosynthesis, and more than 30 genes are involved in the biosynthesis through oxygen-dependent (aerobic) and oxygen-independent (anaerobic) pathways (9). The aerobic pathway of *Pseudomonas denitrificans* has been studied intensively, and the anaerobic pathway has been studied thoroughly in several bacteria, such as *Salmonella typhimurium* and *Bacillus megaterium* (9). These studies have targeted mainly the identification of cobalamin biosynthetic genes and the functions of their encoded enzymes. Genetic engineering techniques involving these genes have also been employed to produce cobalamin (9); however, there has been little discussion about the diversity and phylogeny of cobalamin biosynthetic genes among bacteria, although functional gene diversity is one of the main current issues in microbial ecology (2, 4, 6, 11, 17). In the present study, we tried (i) to screen for cobalamin-producing bacteria, (ii) to reveal the composition of cobalamin biosynthetic genes (*cob* genes) of one of the obtained cobalamin producers, and (iii) to compare the gene arrangement and sequences with other bacteria, so that light could be shed on the phylogeny of the *cob* genes and their relationship with the evolutionary phylogenetic background.

Cobalamin is an essential growth factor for most marine eukaryotic algae, and Croft *et al.* (3) isolated a cobalamin-producing bacterium, *Halomonas* sp., from a nonaxenic culture of the marine microalga *Amphidinium operculatum* (*Dinophyta*). On the other hand, we previously isolated bacteria from nonaxenic cultures of freshwater *Chlorella* (*Chlorophyta*) (14). We expected cobalamin producers to be included within the isolates, taking into account the report by Croft *et al.* (3). In order to screen for cobalamin-producing bacteria from the isolates, we first screened for cobalamin auxotrophic freshwater algae, and revealed that *Monomastix minuta* NIES-255 and NIES-256 (*Chlorophyta*) are cobalamin auxotrophs (see Supplemental material). Using the former strain, we successfully obtained two potential cobalamin producers, *B. megaterium* CSBb and *Ensifer adhaerens* (= *Sinorhizobium morelense*) CSBa (see Supplemental material). The *cob* genes of the former has already been reported (9) but those of the latter have not yet been examined; therefore, we selected *E. adhaerens* CSBa as the material in the subsequent experiments. Additionally, the production of cobalamin by *E. adhaerens* CSBa was confirmed by a brief test (see Supplemental material).

The *cob* genes of *E. adhaerens* CSBa were analyzed as follows. *E. adhaerens* CSBa was cultivated on 1.5% agar-gel medium of 10-fold diluted Bacto Nutrient Broth (Difco, Detroit, MI, USA) at 26°C for 1 week, and the colonies were collected from the medium and suspended in a 1.5-mL microtube containing 1 mL sterile distilled water. The cell suspension was centrifuged at 10,000 rpm for 10 min and the supernatant was removed. The pellet of bacterial cells was then subjected to DNA extraction and purification using ISOIL (Nippon Gene, Tokyo, Japan). Thirty micrograms of the obtained DNA was subjected to pyrosequencing commercially carried out by Hokkaido System Science (Sapporo, Hokkaido, Japan) with a 454 GS-FLX (Roche) by which 575,172 sequences (225,813,225 bp nucleotides) were generated. Each sequence was assembled using GS De novo Assembler software, and a draft genome containing 76 contigs was produced. *cob* Genes were searched from the obtained sequences by BLAST (1), which revealed that three regions contain sequences similar to those of *cob* genes. After start/stop codons were detected on the nucleotide sequences of the three regions using the gene prediction software MetaGene Annotater, the open reading frames (ORFs) highly homologous to *cob* genes were specified. The nucleotide sequences of the ORFs were translated to amino acid sequences with BioRuby (5). The obtained amino acid sequences were subjected to BLAST and Conserved Domain Search (8) using the database in GenBank, and the *cob* genes of *E. adhaerens* CSBa were identified. In addition, a neighbor-joining (NJ) tree was constructed based on the *cobN* sequence by the method described previously (16).

It was revealed that *E. adhaerens* CSBa has 22 *cob* genes: *cobA*, *cobB*, *cobC*, *cobD*, *cobE*, *cobF*, *cobG*, *cobH*, *cobI*, *cobJ*, *cobK*, *cobL*, *cobM*, *cobN*, *cobO*, *cobP*, *cobQ*, *cobS*, *cobT*, *cobU*, *cobV*, and *cobW* (accession numbers, AB705623 to AB705625); this composition of *cob* genes is the same as that of *P. denitrificans* SC510 (9). Recently, the increasing availability of whole genome sequences for a considerable number of bacteria is revealing that many bacteria possess at least some of these 22 genes in the aerobic pathway, regardless of whether they are known as cobalamin biosynthesizing bacteria (cf. Table S1). Other members of the genus *Ensifer*, such as *Ensifer meliloti* (= *Sinorhizobium meliloti*) 1021, *Ensifer medicae* (= *Sinorhizobium medicae*) WSM419, and *Ensifer fredii* (= *Sinorhizobium fredii*) NGR234, are examples that have the same 22 genes (cf. Fig. S1). *Ensifer* species are potential nodule-forming bacteria, and it was demonstrated that *E. meliloti* requires cobalamin-dependent ribonucleotide reductase for symbiosis with its plant host (12). Some other nodule-forming *Rhizobiales* bacteria, such as *Rhizobium leguminosarum* WSM2304, also have these 22 genes (cf. Fig. S1). It is therefore possible to speculate that nodule-forming *Rhizobiales* bacteria generally, if not always, have cobalamin biosynthesis ability. Many bacteria possess both cobalamin-dependent and -independent isozymes. Campbell *et al.* (2) suggested that cobalamin-dependent isozymes likely confer some advantage under specific environmental conditions, and that these isozymes may be important for the survival of *E. meliloti* within a plant host. Not only *E. adhaerens* CSBa but also other members belonging to nodule-forming *Rhizobiales* bacteria have been detected in algal-bacterial consortia (13). It is possible that cobalamin biosynthesis ability might be a key to the symbiotic associations between them. In addition to the above *cob* genes, it is known that some other genes are necessary for the synthesis of cobalamin: *bluB* is one of the genes required for cobalamin biosynthesis in *E. meliloti* (10). The present study revealed that *E. adhaerens* CSBa also has this gene (accession number, AB705624).

The 22 *cob* genes of *E. adhaerens* CSBa are located in three regions (regions 1 to 3), one of which is separated into three subregions (3a to 3c) by multiple ORFs (Fig. 1), forming a tight cluster (operon) within each region or subregion. The above four reference nodule-forming bacteria, *i.e.*, *E. meliloti* 1021, *E. medicae* WSM419, *E. fredii* NGR234, and *R. leguminosarum* WSM2304, have similar *cob* gene operons to those of *E. adhaerens* CSBa (Fig. 1, S1). These bacteria show a high degree of similarity: every pair of two adjacent genes of *cobH*, *cobI*, *cobJ*, *cobK*, and *cobL*, and that of *cobA*, *cobB*, *cobC*, overlaps, except that *E. medicae* WSM419 and *E. fredii* NGR234 have no overlapping between *cobB* and *cobC*.

*Pseudomonas putida* S16 has 18 out of the 22 *cob* genes, but the gene order and/or overlapping pattern in each region are different from those of the above *Rhizobiales* bacteria (Fig. S1). On the other hand, *P. denitrificans* SC510 possesses the 22 *cob* genes, which was revealed not by whole genome analysis but by cloning five operons containing the genes (accession numbers M59236, M59301, M62866, M62868, and M62869). The composition, order, and overlapping pattern of the *cob* genes within the five operons of *P. denitrificans* SC510 are exactly the same as those within region 1, region 2, and subregions 3a to 3c of *E. adhaerens* CSBa (cf. Fig. 1). In addition, both *E. adhaerens* CSBa and *P. denitrificans* SC510 have an ORF homologous to the gene of the histidine-rich transporter transmembrane protein between *cobP* and *cobQ* (cf. Fig. 1), although *E. meliloti* 1021 and *E. medicae* WSM419 have the homologue in a different position distant from *cob* operons.

The nucleotide sequences of the *cob* genes of *E. adhaerens* CSBa were most similar to those of *P. denitrificans* SC510 out of all sequences registered in the DDBJ/EMBL/GenBank databases. The average similarity values between a *cob* gene of *E. adhaerens* CSBa and the corresponding genes of the three other *Ensifer* species ranged from 65.4 to 92.1% (78.3% on average); however, those of *E. adhaerens* CSBa and *P. denitrificans* SC510 ranged as high as from 96.6 to 99.8% (98.9% on average) (Table S2).

In the neighbor-joining tree based on the *cobN* sequence (*ca.* 3,800–4,000 bp), which is the longest of the 22 *cob* genes, the possessors of *cobN* were separated into four clusters with high bootstrap values, generally reflecting taxonomy to some extent, that is, the phylogeny of *cobN* is basically congruent with that of the 16S rRNA gene (Fig. 2). Clusters I, II, III, and IV generally correspond to the classes *Alphaproteobacteria*, *Betaproteobacteria*, *Gammaproteobacteria*, and the phylum *Actinobacteria*, respectively. The four *Ensifer* species formed a cluster together, clearly separated from the other two genera, *Rhizobium* and *Agrobacterium*, of the same family *Rhizobiaceae*.

Discrepancies between *cobN* phylogeny and taxonomy were recognized for *Ralstonia solanacearum* (*Betaproteobacteria*) GMI1000 and *P. denitrificans* (*Gammaproteobacteria*) SC510 in the NJ tree (Fig. 2). The position of the latter is very close to that of *E. adhaerens* CSBa, which is a reflection of the high sequence similarities of the *cob* genes described above. Based on the NJ-tree topology, it is fair to assume that the *cobN* of *E. adhaerens* CSBa might be inherited from common ancestors of the four *Ensifer* members; however, *cobN* of *P. denitrificans* SC510 is distant from those of other *Pseudomonas* members but within the *Ensifer* cluster close to *E. adhaerens* CSBa. The other 21 *cob* genes of *P. denitrificans* SC510 also showed the same phylogenetic relationship to *E. adhaerens* CSBa (data not shown). The above NJ-tree topology, as well as the high degree of similarity in the *cob* gene arrangement described above, suggests that an ancestor of *P. denitrificans* SC510 obtained the 22 *cob* genes from an *Ensifer* bacterium phylogenetically closely related to *E. adhaerens* CSBa, perhaps by lateral gene transfer. Krishnapillai (7) named the *cob* operon as one of the prokaryotic DNA segments postulated to be involved in lateral gene transfer, since the *cob* operons in *Escherichia coli* and *S. typhimurium* had a higher GC content (59%) than the genome average of 52%. The average GC content of the 22 *cob* genes of *E. adhaerens* CSBa and *P. denitrificans* SC510 is approximately 66%, and that of the genomes of *Ensifer* species and *P. denitrificans* is approximately 62–63% each, which unfortunately does not help to elucidate the validity of the above suggestion in the present study. *P. denitrificans* SC510 and its mutants have been well studied and used for the commercial production of cobalamin for decades. It was somewhat surprising that we stumbled on the possible origin of the *cob* genes of *P. denitrificans* SC510 in a *Rhizobiales* bacterium.

In the present study, we revealed that *E. adhaerens* CSBa is a cobalamin producer and has 22 *cob* genes. It was suggested that *cob* gene phylogeny is generally congruent with that of the 16S rRNA gene, but the genes can be laterally transferred. Bertrand *et al.* (1) suggested that a previously undescribed group of bacteria could dominate the B_12_ biosynthesizing community in a certain environment, indicating the potential benefits of *cob* genes as a tool for ecological studies. The above congruency presented here would be valuable as a guide to the further study of the diversity of cobalamin producers and the distribution of cobalamin-dependent events in microbial ecology.

The nucleotide sequences determined in this study have been deposited in the DDBJ/EMBL/GenBank and assigned accession numbers AB705623–AB705625.

## Supplementary Material



## Figures and Tables

**Fig. 1:**
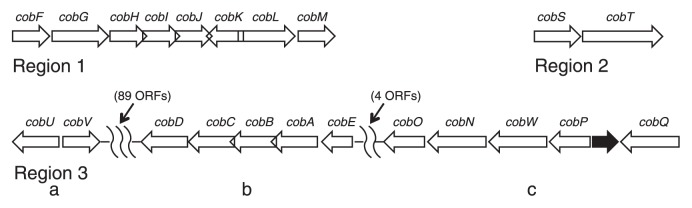
Arrangement of *cob* genes of *Ensifer adhaerens* CSBa identified in the present study. The *cob* genes are depicted by thick open arrows, which indicate the direction of transcription. A thick closed arrow is an ORF homologous to the gene of histidine-rich transporter transmembrane protein. The points where arrows overlap (between every pair of two adjacent genes of *cobH*, *cobI*, *cobJ*, *cobK*, and *cobL*, and of *cobA*, *cobB*, *cobC*) indicate that the stop/start codons of these genes overlap. The order and overlapping pattern of the genes within each of region 1, region 2, and subregions 3a to 3c are the same as those of *Pseudomonas denitrificans* SC510.

**Fig. 2:**
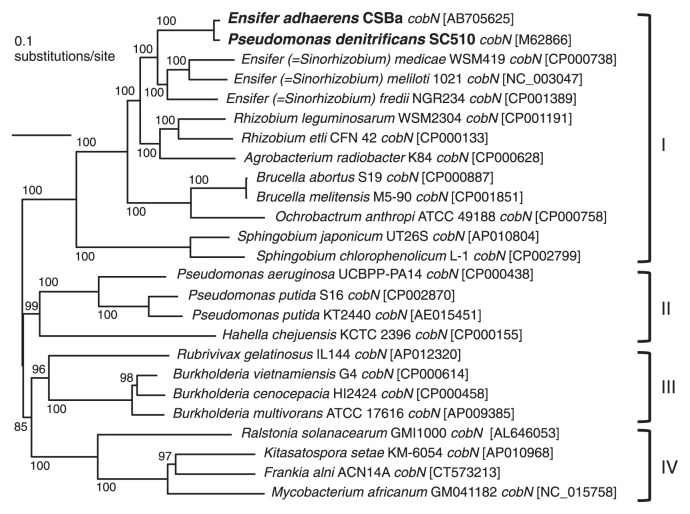
Phylogenetic tree based on the *cobN* sequence of *Ensifer adhaerens* CSBa and other bacteria constructed by the neighbor-joining method. Bootstrap values, indicated at the nodes, were calculated from 100 resamplings. Accession numbers in DDBJ/EMBL/GenBank are shown in brackets. Clusters I, II, III, and IV generally correspond to the classes *Alphaproteobacteria*, *Betaproteobacteria*, *Gammaproteobacteria*, and the phylum *Actinobacteria*.
