# Supplementary material for: Identification and Phylogenetic Characterization of Cobalamin Biosynthetic Genes of Ensifer adhaerens
Source: Microbes Environ. 2012 Dec 19;28(1):153–5. doi: 10.1264/jsme2.ME12069 (PMC4070679; doi:10.1264/jsme2.ME12069)
Supplement: Supplementary file 1 [file 28_153_s1.pdf]

Supplemental Materials

Identification and phylogenetic characterization of cobalamin biosynthetic genes of *Ensifer adhaerens*

Hoan Thi Vu[1], Hideomi Itoh[1], Satoshi Ishii[1] †, Keishi Senoo[1], and Shigeto Otsuka[1]*

[1] *Department of Applied Biological Chemistry, Graduate School of Agricultural and Life Sciences, The University of Tokyo, 1-1-1 Yayoi, Bunkyo-ku, Tokyo 113-8657, Japan*

† **Present address**: Division of Environmental Engineering, Faculty of Engineering, Hokkaido University, North 13, West 8, Kita-ku, Sapporo, Hokkaido 060-8628, Japan.

Materials and Methods

Screening of cobalamin-auxotrophic freshwater algae

The following 12 axenic cultures of freshwater green microalgae were obtained from the Microbial Culture Collection at the National Institute for Environmental Studies (MCC-NIES), Tsukuba, Japan: *Chlorella vulgaris* NIES-227, *C. vulgaris* NIES-2170, *C. vulgaris* NIES-2172, *C. vulgaris* NIES-641, *Chlorella sorokiniana* NIES-2167, *C. sorokiniana* NIES-2168, *C. sorokiniana* NIES-2169, *Chlorella elkhatiense* NIES-2250, *Chlorella* sp. NIES-2171, *Chlorella* sp. NIES-2330, *Monomastix minuta* NIES-255, and *M. minuta* NIES-256. *Chlorella* species and *M. minuta* belong to the classes *Trebouxiophyceae* and *Prasinophyceae* of the phylum *Chlorophyta*, respectively. These algae were maintained in C medium (2) except for *M. minuta* NIES-256 maintained in AF-6 medium (3), under a 16:8 light/dark cycle with a light intensity of approximately 20 μmol photons m^{-2} sec^{-1} at about 25°C in light and 20°C in dark. C and AF-6 media contain cobalamin. We also prepared cobalamin-free C and AF-6 media, and transferred 500 μL of the culture fluid of each alga to fresh C or AF-6 medium with and without cobalamin in triplicate. These algae were cultivated under the same conditions as described above. Once every two weeks, all the cultures were subcultivated, and this was repeated five times. At any point during this test, a strain was regarded as cobalamin auxotrophic when it died in the medium without cobalamin and remained alive in the medium with cobalamin.

Screening of bacteria that provide cobalamin to algae

Bacteria that is expected to provide cobalamin to algae were screened from eight strains: *Variovorax* sp. C6d, *Ensifer adhaerens* CSBa, *Bacillus megaterium* CSBb, *Microbacterium testaceum* CSBd, *Caulobacter* sp. C2a1, *Emticicia ginsengisoli* C2b, *Polaromonas* sp. C2d, and *Brevundimonas* sp. C2e1. These were isolated in our previous study from consortia of microalgae and soil bacteria (4) and were maintained on 1.5% agar-gelled medium of 10-fold-diluted Bacto Nutrient Broth (Difco) (hereafter referred to as 1/10 NB agar medium) at 26°C. Two to five colonies of each bacterial strain were collected from the medium (the number of colonies depended on the colony size) 3 to 7 days after the last subcultivation and transferred to 1 ml of C medium. The cell suspension was centrifuged at 5,000 ×g for 15 min and resuspended in 1 ml of C medium. This was repeated twice to remove the culture broth.

M. minuta NIES-255 was selected as a cobalamin-auxotrophic alga according to the result of the screening of cobalamin-auxotrophic algae (described later). Two milliliters of *M. minuta* NIES-255 culture fluids at 2 weeks after the last subcultivation was transferred to a 2-ml microtube and centrifuged at 2,000 ×g for 5 min. After the removal of the supernatant to remove cobalamin, the cell pellet was suspended in 1 ml of C medium without cobalamin and centrifuged again. This step was repeated twice. Then the algal cells were resuspended in 1 ml of C medium without cobalamin.

The above-prepared cell suspensions of each bacterial strain and *M. minuta* NIES-255 were transferred to 90 ml of fresh C medium with and without cobalamin in a 200-ml glass flask capped with a Plug Silicon (AZ One, Osaka, Japan). This co-culture was prepared in triplicate for each bacterial strain. In addition, three control cultures of the alga without bacteria were prepared. The co-cultures and control cultures were cultivated under the same growth conditions for the alga as described above for 8 weeks, and the algal growth was checked.

Results

Out of the 12 freshwater green microalgal strains, 10 strains of *Chlorella* grew without cobalamin, and *M. minuta* NIES-255 and NIES-256 could not grow without cobalamin. Croft et al. (1) compiled the cobalamin auxotrophy of algae reported in the literature, in which 20 *Chlorella* species, including *C. vulgaris* and *C. sorokiniana*, were regarded as cobalamin non-auxotrophic. We thought it possible that even one algal species could include both cobalamin-auxotrophic and non-auxotrophic members, because the relationship between auxotrophy and the species delimitation has not been clear. However, all the *Chlorella* strains examined in the present study turned out to be cobalamin non-auxotrophic.

Cobalamin-auxotrophic alga *M. minuta* NIES-255 could grow in cobalamin-free C medium in the presence of *E. adhaerens* CSBa and *B. megaterium* CSBb, indicating the

possibility that these two could produce cobalamin and release it into the medium. The cobalamin production by *E. adhaerens* CSBa was confirmed by using Vitamin B_{12} Assay Kit (Immundiagnostik GmbH, Bensheim, Germany) according to manufacturer's protocol. The concentration of cobalamin produced by this strain was at least 80 ng/l in a medium containing 0.1% glucose, 0.1% NH_4NO_3, 0.1% KH_2PO_4, 0.1% $MgSO_4.7H_2O$, 0.1% NaCl, and 0.1% L-glutamic acid (pH 7.0), after a week of cultivation at 25°C.

References

1. Croft, M.T., A.D. Lawrence, E. Raux-Deery, M.J. Warren, and A.G. Smith. 2005. Algae acquire vitamin B_{12} through a symbiotic relationship with bacteria. Nature. 483:90–93.
2. Ichimura, T. 1971. Sexual cell division and conjugation-papilla formation in sexual reproduction of *Closterium strigosum*, p. 208–214. *In* K. Nishizawa (ed.), Proceedings of the 7th International Seaweed Symposium. University of Tokyo Press, Tokyo.
3. Kato, S. 1982. Laboratory culture and morphology of *Colacium vesiculosum* Ehrb. (*Euglenophyceae*). Jpn. J. Phycol. 30:63–67.
4. Vu, H.T., S. Otsuka, H. Ueda, and K. 2010. Cocultivated bacteria can increased or decreased the culture lifetime of *Chlorella vulgaris*. J. Gen. Appl. Microbiol. 56:413–418.

Table S1. Distribution of putative *cob* genes among bacteria based on genomic information.

Class	Species and strain[1]	*cob* gene[2]																					
		A	*B*	*C*	*D*	*E*	*F*	*G*	*H*	*I*	*J*	*K*	*L*	*M*	*N*	*O*	*P*	*Q*	*S*	*T*	*U*	*V*	*W*
Alpha-proteobacteria	*Ensifer* spp.	+	+	+	+	+	+	+	+	+	+	+	+	+	+	+	+	+	+	+	+	+	+
	Rhizobium leguminosarum WSM2304	+	+	+	+	+	+	+	+	+	+	+	+	+	+	+	+	+	+	+	+	+	+
	Mesorhizobium loti MAFF303099	+	+	+	+	+	+	+	+	+	+	+	+	+	+	+	+	+	+	+	+	–	+
	Sphingobium japonicum UT26S	+	+	+	+	–	+	+	+	+	+	+	+	+	+	+	+	+	+	+	+	–	+
Beta-proteobacteria	*Burkholderia multivorans* ATCC 17616	–	+	+	+	–	–	+	+	+	+	+	+	+	+	+	+	+	+	+	+	+	+
	Rubrivivax gelatinosus IL144	+	+	+	+	–	+	–	+	+	+	+	+	+	+	+	–	+	+	+	+	–	+
	Ralstonia solanacearum GMI1000	+	+	+	+	–	–	–	+	–	–	–	+	–	+	–	+	+	+	+	–	–	+
Gamma-proteobacteria	*Pseudomonas denitrificans* SC510	+	+	+	+	+	+	+	+	+	+	+	+	+	+	+	+	+	+	+	+	+	+
	Pseudomonas putida S16	+	+	+	+	+	+	+	+	+	+	+	+	+	+	+	+	+	–	–	–	–	+
	Pseudomonas aeruginosa PAO1	+	+	+	+	–	+	–	+	+	+	+	+	+	+	+	+	+	–	–	+	+	+
	Hahella chejuensis KCTC 2396	–	–	–	+	–	–	–	+	+	+	+	+	+	+	+	–	+	+	+	+	–	–
Actinobacteria	*Frankia alni* ACN14A	–	+	–	+	+	–	+	+	+	–	+	+	+	+	+	–	+	+	+	+	–	–
	Kitasatospora setae KM–6054	+	+	+	+	–	–	+	+	+	+	+	+	+	+	+	–	+	+	+	+	–	–
	Mycobacterium africanum GM041182	–	+	+	+	–	–	+	+	+	–	+	+	+	+	+	–	+	+	+	+	–	–

[1] *Ensifer* spp. includes *Ensifer adhaerens* CSBa, *Ensifer meliloti* 1021, *Ensifer medicae* WSM419, and *Ensifer fredii* NGR234.

[2] Accession numbers in DDBJ/EMBL/GenBank database are as follows: AB705623 to AB705625 (*E. adhaerens* CSBa); NC_003047 (*E. meliloti* 1021); CP000738 (*E. medicae* WSM419); CP001389 (*E. fredii* NGR234); CP001191, CP001192, and CP001193 (*R. leguminosarum* WSM2304); NC_002678 (*M. loti* MAFF303099); AP010803 and AP010804 (*S. japonicum* UT26S); AP009385 to AP009388 (*B. multivorans* ATCC 17616); AP012320 (*R. gelatinosus* IL144); NC_003295 and AL646053 (*R. solanacearum* GMI1000); M59236, M59301, M62866, M62868, and M62869 (*P. denitrificans* SC510); CP002870 (*P. putida* S16); NC_002516 (*P. aeruginosa* PAO1). CP000155 (*H. chejuensis* KCTC 2396); CT573213 (*F. alni* ACN14A); AP010968 (*K. setae* KM-6054); and NC_015758 (*M. africanum* GM041182).

Table S2. Sequence similarities (%) of *cob* genes between *Ensifer adhaerens* CSBa and four other bacteria.

Gene	*Ensifer meliloti* 1021	*Ensifer medicae* WSM419	*Ensifer fredii* NGR234	*Pseudomonas denitrificans* SC510
cobA	81.7	80.3	83.1	99.4
cobB	74.3	72.2	75.5	99.1
cobC	68.4	68.3	69.1	99.3
cobD	73.8	70.0	74.0	98.9
cobE	71.5	70.8	69.1	99.5
cobF	80.4	77.1	80.4	99.0
cobG	67.7	65.4	66.1	99.0
cobH	82.9	80.3	84.5	99.8
cobI	80.1	78.5	81.5	99.6
cobJ	79.1	77.5	77.1	98.8
cobK	74.0	71.5	74.4	99.1
cobL	75.9	74.0	76.2	96.6
cobM	83.8	82.2	83.8	98.7
cobN	79.0	77.8	77.6	98.3
cobO	85.1	84.2	87.1	98.9
cobP	78.2	76.7	77.6	99.2
cobQ	80.4	77.7	80.6	98.4
cobS	91.0	90.0	92.1	99.6
cobT	84.9	83.3	85.1	98.5
cobU	85.7	84.0	87.3	99.2
cobV	70.8	70.7	73.4	99.0
cobW	84.6	82.6	86.1	98.1

Supplemental Figure legend

Fig. S1. Arrangement of *cob* genes of *Ensifer meliloti* 1021, *Ensifer medicae* WSM419, *Ensifer fredii* NGR234, *Rhizobium leguminosarum* WSM2304, and *Pseudomonas putida* S16, depicted based on the sequence and annotation information on the genomes and plasmids of these bacteria (accession numbers in DDBJ/EMBL/GenBank, NC_003047, CP000738, CP001389, CP001191 to CP001193, and CP002870, respectively). The *cob* genes are depicted by thick open arrows, which indicate the direction of transcription. Thick closed arrows are non-*cob* ORFs. More than three successive ORFs are indicated by undulating lines. The points where arrows overlap indicate that the stop/start codons of these genes overlap each other. The *cob* genes of *E. meliloti* 1021, *E. medicae* WSM419, *E. fredii* NGR234, and *R. leguminosarum* WSM2304 are located in three regions, and those of *P. putida* S16 are located in at least five regions.

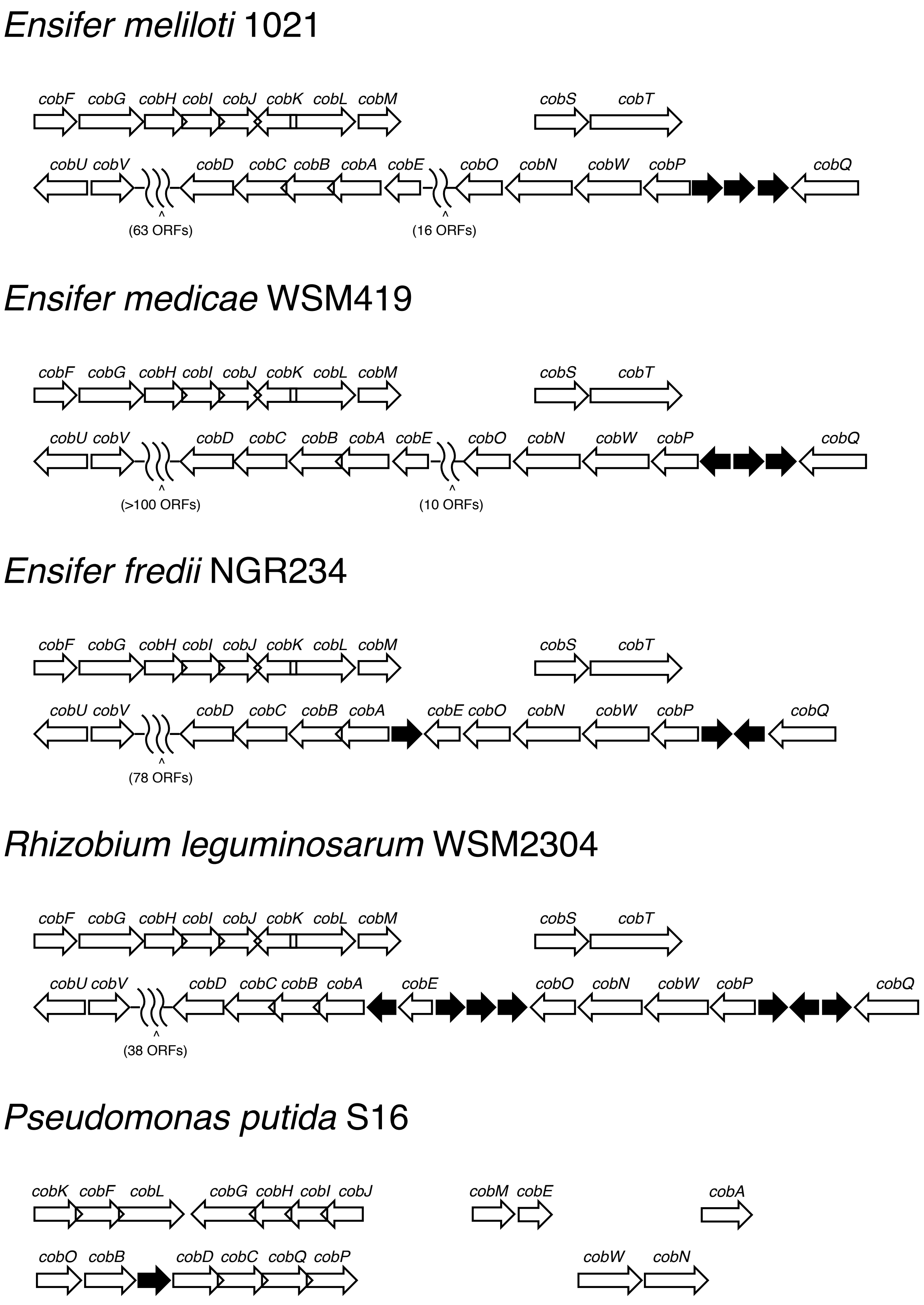

Fig. S1. Arrangement of *cob* genes in reference species/strains.
